# Associations of residential green space with internalizing and externalizing behavior in early childhood

**DOI:** 10.1186/s12940-024-01051-9

**Published:** 2024-02-08

**Authors:** Marnie F. Hazlehurst, Anjum Hajat, Pooja S. Tandon, Adam A. Szpiro, Joel D. Kaufman, Frances A. Tylavsky, Marion E. Hare, Sheela Sathyanarayana, Christine T. Loftus, Kaja Z. LeWinn, Nicole R. Bush, Catherine J. Karr

**Affiliations:** 1 Department of Epidemiology, Department of Environmental & Occupational Health Sciences, University of Washington School of Public Health, 4225 Roosevelt Way NE, Seattle, WA 98105 USA; 2 Department of Epidemiology, University of Washington School of Public Health, Seattle, WA USA; 3 Seattle Children’s Research Institute, Department of Pediatrics, University of Washington School of Medicine, Seattle, WA USA; 4 Department of Biostatistics, University of Washington School of Public Health, Seattle, WA USA; 5 Department of Environmental & Occupational Health Sciences, Department of Epidemiology, Division of General Internal Medicine, Department of Medicine, University of Washington School of Public Health, University of Washington School of Medicine, Seattle, WA USA; 6 Department of Preventive Medicine, University of Tennessee Health Science Center, Memphis, TN USA; 7 Seattle Children’s Research Institute; Department of Pediatrics, University of Washington School of Medicine; Department of Environmental & Occupational Health Sciences, University of Washington School of Public Health, Seattle, WA USA; 8 Department of Environmental & Occupational Health Sciences, University of Washington School of Public Health, Seattle, WA USA; 9 Department of Psychiatry School of Medicine, University of California San Francisco, San Francisco, CA USA; 10 Department of Psychiatry, Department of Pediatrics, School of Medicine, University of California San Francisco, San Francisco, CA USA; 11 Department of Pediatrics, Department of Environmental & Occupational Health Sciences, University of Washington School of Medicine, University of Washington School of Public Health, Seattle, WA USA

**Keywords:** Child mental health, Internalizing, Externalizing, Built environment, Green space

## Abstract

**Background:**

Green space exposures may promote child mental health and well-being across multiple domains and stages of development. The aim of this study was to investigate associations between residential green space exposures and child mental and behavioral health at age 4–6 years.

**Methods:**

Children’s internalizing and externalizing behaviors in the Conditions Affecting Neurocognitive Development and Learning in Early Childhood (CANDLE) cohort in Shelby County, Tennessee, were parent-reported on the Child Behavior Checklist (CBCL). We examined three exposures—residential surrounding greenness calculated as the Normalized Difference Vegetation Index (NDVI), tree cover, and park proximity—averaged across the residential history for the year prior to outcome assessment. Linear regression models were adjusted for individual, household, and neighborhood-level confounders across multiple domains. Effect modification by neighborhood socioeconomic conditions was explored using multiplicative interaction terms.

**Results:**

Children were on average 4.2 years (range 3.8-6.0) at outcome assessment. Among CANDLE mothers, 65% self-identified as Black, 29% as White, and 6% as another or multiple races; 41% had at least a college degree. Higher residential surrounding greenness was associated with lower internalizing behavior scores (-0.66 per 0.1 unit higher NDVI; 95% CI: -1.26, -0.07) in fully-adjusted models. The association between tree cover and internalizing behavior was in the hypothesized direction but confidence intervals included the null (-0.29 per 10% higher tree cover; 95% CI: -0.62, 0.04). No associations were observed between park proximity and internalizing behavior. We did not find any associations with externalizing behaviors or the attention problems subscale. Estimates were larger in neighborhoods with lower socioeconomic opportunity, but interaction terms were not statistically significant.

**Conclusions:**

Our findings add to the accumulating evidence of the importance of residential green space for the prevention of internalizing problems among young children. This research suggests the prioritization of urban green spaces as a resource for child mental health.

**Supplementary Information:**

The online version contains supplementary material available at 10.1186/s12940-024-01051-9.

## Background

The residential neighborhood context has been identified as an important contributor to behavioral and mental health and development [1–3]. Natural environments in particular may promote healthy development for children, even early in childhood [4]. Given the plasticity of the developing brain, early life may be an especially important window for these exposures [5, 6]. This early developmental window is of further interest as the onset of externalizing and internalizing behavior problems in early life are linked to poorer outcomes in several domains later in life. Externalizing behaviors, characterized by behavior directed outwards at an individual’s environment, include attention problems, rule-breaking, and aggressive behavior [7]; may interfere with functioning in school and social settings; and are associated with further externalizing behaviors in adolescence, as well as substance use disorders, lower academic achievement, accidental injury, and obesity across the life course [8]. Behavior problems in the internalizing domain include behaviors directed towards the self, such as depression, anxiety, and social withdrawal [9]. Internalizing symptoms can also emerge in early life and are associated with subsequent internalizing problems in adolescence and adulthood, substance use, and other significant functional impairments.

The evidence to date suggests an inverse relationship between green space and behavior problems in childhood, particularly for externalizing behaviors [10]. In early school-age children, research on green space and behavior has primarily focused on attention, including Attention Deficit/Hyperactivity Disorder (ADHD) symptoms and diagnosis, working memory, and attentional and inhibitory control [11–13]. Prior studies are also suggestive of an association between green space and internalizing behavior, though results are generally less consistent than those for the externalizing domain. Among school-age children and adolescents, studies of residential green space have observed associations of higher green space with reduced symptoms of depression or anxiety [14–16]. Though internalizing behavior problems can emerge early in childhood, few studies of green space have focused on outcomes in this domain at these younger ages.

Natural environments may affect child behavioral and mental health through multiple mechanisms. Exposures to green space, including public green space, may promote child physical activity and free play, facilitate social connections, or reduce adverse environmental exposures [17, 18]. Some of these mechanisms may be operating at the neighborhood scale or require time spent playing in green spaces. Exposures may also directly influence cognitive processing or psychophysiological mechanisms. Prior observational and experimental studies have largely been informed by Attention Restoration Theory (ART) or Stress Recovery Theory (SRT). ART suggests that natural environments facilitate recovery of cognitive processing resources by allowing for restoration of directed attention [19–21]. This hypothesized mechanism has motivated the focus on attention outcomes in children. However, SRT suggests that contact with nature may also directly impact perceived stress and physiological measures of the stress response, including regulation of autonomic nervous system activity and the hypothalamic–pituitary–adrenal (HPA) axis [22]. Overall greenness in the immediate neighborhood and specific green space forms such as tree canopy, may be particularly relevant for these mechanisms [23].

Though there is a rapidly accumulating literature on the relationship between green space and behavioral and mental health, several limitations of this literature have been identified [4, 10, 24]. Some studies have been limited to a single residential location at a single time point or only considered one green space exposure measure; relatively few studies have examined exposures in the first years of life or included multiple green space indicators. A number of prior studies have been unable to adjust for potentially important confounders, including measures such as maternal depression or neighborhood socioeconomic characteristics. Studies with large sample sizes have also often been limited to a categorical diagnosis to assess behavioral and mental health outcomes or have used study populations of older children.

We investigated the relationships between residential green space exposures and child behavioral and mental health within the Conditions Affecting Neurocognitive Development and Learning in Early Childhood (CANDLE) study. Specifically, we examined both internalizing and externalizing behaviors at age 4–6 in this well-characterized, socioeconomically and racially diverse cohort. We explored effect modification by neighborhood socioeconomic opportunity and by child sex, and conducted a number of sensitivity analyses to assess the robustness of modeled relationships.

## Methods

### Study population

The CANDLE study is a longitudinal pregnancy cohort located in Shelby County, TN [25, 26]. This observational cohort is one of three cohort studies within the ECHO-PATHWAYS consortium [27]. The CANDLE study was established specifically to investigate determinants of child neurodevelopment. Pregnant women (*N* = 1503) were enrolled in CANDLE between 2006 and 2011 (births in 2007–2011). Women were excluded from participation in the CANDLE study if they were considered to have a high medical risk pregnancy, which was defined as having an existing chronic disease requiring medication or a known pregnancy complication at the time of recruitment. Each woman provided informed consent upon enrollment. When children were between ages 4 and 6, CANDLE mothers completed a suite of surveys, and mother-child dyads attended an in-person study visit. The analytic sample was restricted to those who reported a current address within Shelby County, TN at the time of the study visit or who reported a residential history within Shelby County for at least 75% of the year prior to the study visit in order to assess residential green space exposures. The CANDLE study was approved by the University of Tennessee Health Science Center Institutional Review Board (IRB). The current analysis was conducted by ECHO-PATHWAYS and was approved by the University of Washington IRB.

### Child behavior

The preschool (1.5-5 years) version of the Achenbach System of Empirically Based Assessment Child Behavior Checklist (CBCL) was administered at the age 4–6 study visit for children < 6 years old [28]. Mothers reported “not true” (coded as 0), “somewhat or sometimes true” (coded as 1), or “very true or often true” (coded as 2), for 99 child behaviors. Each question was asked in reference to the two months prior to the study visit and missing responses were treated as zeroes. Responses to these individual items were summed to calculate the broadband scores and related syndrome subscales. Assessment of child behavior in early life commonly relies upon parent-report to evaluate behavior across multiple contexts. A particular strength of this tool is that it includes a wide range of behaviors. The CBCL has been validated for use in community samples and is widely used in both clinical and research settings [7].

The primary outcomes of interest in this analysis were the broadband internalizing score, the broadband externalizing score, and the attention problems subscale. At the preschool age, the attention problems syndrome scale contributes to the broadband externalizing score and has shown diagnostic value in screening for ADHD [29]. In secondary analyses, we explored the four syndrome scales in the internalizing domain: the emotionally reactive, anxious/depressed, somatic complaints, and withdrawn scales, as well as the other subscale in the externalizing domain: aggressive behavior. In both primary and secondary analyses, we used raw scores modeled as discrete variables. For descriptive purposes only, CBCL scores were dichotomized at clinical and borderline-clinical thresholds, defined as a t-score greater than 63 or a t-score greater than or equal to 60, respectively.

### Green space

In this study, we examined three distinct measures of exposure to green space. First, the Normalized Difference Vegetation Index (NDVI) was used to assess the overall greenness of the area surrounding the residential location. We calculated NDVI at each participant residence using 2011 annual data at 30 m resolution from the NASA Global Web-Enabled Landsat Data (GWELD) [30]. Water (indicated by pixels with a value less than zero) was excluded, resulting in a scale ranging from 0 to 1 in each pixel of the dataset. We considered average NDVI within a 300 m buffer weighted by the residential history across the year prior to the age 4–6 study visit as the primary exposure. NDVI weighted by the residential history from age 1 to age 4 was explored in a secondary analysis. Using only a single address, at which the child had lived the longest, was explored in a sensitivity analysis. A 300 m buffer was used in the primary analysis because a distance of 300 m has often been used in policy and urban planning, as well as in prior epidemiologic studies, particularly when accessibility to green space is of interest [31]. A smaller buffer likely reflects more private rather than public green space, whereas buffers of 1000 m have been justified based on 5–10 min walking distances for adults; we explored these exposures in secondary analyses [32].

The second exposure measure used in this study was the percent of land area covered by tree canopy. Tree cover data were obtained from the US Environmental Protection Agency (EPA) EnviroAtlas and reflect the percent of the census block group covered by trees in 2008–2013 [33]. This measure includes street trees, parks, urban forests, and single trees on various properties, which were derived from 1 m resolution landcover data and aggregated to the census block group level. Percent tree canopy was weighted by the residential history across the year prior to the age 4–6 study visit in primary analyses; we also explored exposures averaged over the residential history from age 1 to age 4 or at the single longest-lived address.

We also examined a measure of access to green spaces: park proximity to the residence was calculated as Euclidean distance from the home location to the nearest boundary of a park in meters, using data on park boundaries in 2019 compiled by the Trust for Public Land [34]. Distance to the nearest park was calculated for the address at the time of outcome assessment as the primary measure and for the address at which the child lived the longest in a sensitivity analysis. In further sensitivity analyses, we calculated the distance to the nearest small park (< 2 acres), neighborhood park (2–20 acres), and community park (> 20 acres), separately [35]. Unlike the other two green space measures for which a higher value represents greater exposure to natural environments, park proximity was coded such that a lower value represents closer proximity, conceptualized as better access.

### Covariates

An extensive suite of variables has been collected in CANDLE, including both maternal and child information. Maternal education was reported in five categories (less than high school, high school degree, technical school, college degree, or graduate/professional degree). Self-reported maternal race was included as Black, White, or another race/multiple races. Given the history of residential segregation and discrimination in the US, maternal race was considered a proxy measure for access to neighborhood resources and exposure to stressors, as well as a proxy for broader environmental conditions in the community. Household income was reported in 8 categories ($0-$15,000 was the lowest category, each of the next 6 categories were in increments of $10,000, and the highest category was $75,000 or more) at the age 4–6 visit. We converted income to a continuous variable by selecting the midpoint of each category; due to right-censoring, in the highest category the Pareto distribution was used to assign the income level [36]. This continuous income variable was then adjusted for the number of adults and children in the household at the age 4–6 visit using the OECD equivalence scale, which assigns a value of 1 to the first adult in the household and a value of 0.5 for each additional adult and a value of 0.3 for each child in the household [37]. Maternal IQ was assessed using the Wechsler Abbreviated Scale of Intelligence (WASI) first edition, four-subtext form [38, 39] and maternal depression was assessed using the Center for Epidemiological Studies-Depression scale (CES-D) at the age 4–6 study visit [40]; both were included as continuous covariates. The preschool version (parent-report for ages 2–5) of the Parenting Relationship Questionnaire (PRQ) was administered at the age 4–6 visit for all children and the attachment score was used as a continuous variable [41]. Maternal tobacco smoking during pregnancy was defined as either self-report of tobacco use or a urinary cotinine level greater than 200 ng/mL in a 2nd or 3rd trimester sample. Gestational age at birth was obtained from medical records and dichotomized; preterm was defined as a gestational age < 37 weeks. Residential instability was calculated as number of changes of address between the child’s birth to the age 4–6 study visit from reported residential history and specified as a count.

Several child behaviors were also reported at age 4–6 in CANDLE and were included in extended models. Physical activity was reported as how many times in a normal week the child engaged in vigorous physical activity in three categories (never or occasionally, once or twice per week, and three or more times per week). Screen time, specified as including watching television and using a computer, was reported as the number of hours per day.

Address histories were also used to characterize several neighborhood-level conditions. Urbanicity was assessed at the census tract level using census designations based on population density. Neighborhood resources were operationalized using the socioeconomic and education opportunity subscales of the Childhood Opportunity Index (COI) at the census tract level [42]. The socioeconomic scale is comprised of several variables at the census tract level, including poverty rate, homeownership rate, median household income, and employment rate. The education scale includes factors related to early childhood education, elementary education, secondary and post-secondary education, and educational resources. COI was weighted across the same address history as the green space exposure of interest. Both COI-socioeconomic and COI-education subscales were included separately in the models as covariates. In effect modification analyses, we examined the COI socioeconomic subscale as an effect modifier. In extended models, we accounted for living near a major roadway. Distance to the nearest major roadway (class A1, A2, or A3 road) was dichotomized at 150 m to indicate a near-road residence.

### Statistical analysis

Descriptive statistics were calculated for exposures, outcomes, and covariates. We used linear regression with robust standard errors to assess the association between green space and the continuous raw CBCL scores.

We used a staged model approach to covariate adjustment. Covariates were identified *a priori* by reviewing the literature. Model 1 was considered minimally-adjusted, with only child sex and child age at outcome assessment included as covariates in the model. Model 2 was additionally adjusted for socioeconomic status, neighborhood resources, and proxies for socioeconomic resources; specifically, the covariates in model 2 included maternal education, household income adjusted for household size, maternal race, residential stability, COI socioeconomic scale, COI education scale, and urbanicity. Model 3 was considered primary and includes all of the covariates in model 2 as well as further adjustment for some additional factors related to child neurodevelopment, including maternal IQ, maternal depression, PRQ attachment score, maternal smoking during pregnancy, and preterm birth. Sensitivity analyses included a set of extended models, each with further adjustment for another individual or environmental factor, including child physical activity, child screen time, and a residential location near a major roadway. Effect modification by neighborhood SES (COI socioeconomic scale) and child sex was assessed by inclusion of a multiplicative interaction term in the model.

All data manipulations, visualizations, and analysis were conducted in R 3.6 (The R Foundation for Statistical Computing; Vienna, Austria).

## Results

In CANDLE, 1,030 participants completed the CBCL at the age 4–6 study visit. There were 943 children in the final analytic sample with at least one of the three primary green space exposure measures and CBCL scores at age 4–6 (see details of exclusions in Supplemental Fig. 1).

Characteristics of the participants are shown for the analytic sample overall and by quartile of NDVI exposure in Table 1. Approximately half of the sample were boys and the mean age of children at the time of the CBCL assessment was 4.3 years (SD 0.4). Participant characteristics were generally similar to those of Shelby County overall [43]. In this sample, 65% of mothers self-identified as Black, 29% as White, and 6% as another race or multiple races; 41% had a college or graduate/professional degree. The mean internalizing domain raw score was 6.21 (SD 6.17) and T-score was 45 (SD 10.8) (Table 2); 5.3% of children in this sample had an internalizing score above the clinical threshold and 11.0% had an internalizing score above the borderline-clinical threshold. The mean externalizing domain raw score was 9.18 (SD 7.57) and T-score was 44.9 (SD 10.4); 4.8% and 7.7% of children in this sample had an externalizing score above the clinical and borderline-clinical thresholds, respectively. In this sample, 2.2% of children scored above clinical thresholds in both the internalizing and externalizing domains.

**Table 1:** Characteristics of the analytic sample

	Full analytic sample (*n* = 943)		Sample by quartile of NDVI in 300 m							
			Q1 (*n* = 230)		Q2 (*n* = 230)		Q3 (*n* = 229)		Q4 (*n* = 230)	
**Child variables**										
*Categorical*	*n*	*(%)*	*n*	*(%)*	*n*	*(%)*	*n*	*(%)*	*n*	*(%)*
Boys	465	(49)	115	(50)	113	(49)	108	(47)	120	(52)
Girls	478	(51)	115	(50)	117	(51)	121	(53)	110	(48)
Preterm birth	83	(9)	20	(9)	17	(7)	26	(11)	19	(8)
SHS exposure	287	(31)	71	(31)	74	(32)	67	(30)	65	(28)
*Continuous*	*mean*	*(SD)*	*mean*	*(SD)*	*mean*	*(SD)*	*mean*	*(SD)*	*mean*	*(SD)*
Age (yrs)	4.3	(0.4)	4.3	(0.4)	4.3	(0.4)	4.3	(0.4)	4.3	(0.4)
Physical activity	1.6	(0.7)	1.5	(0.8)	1.7	(0.7)	1.6	(0.7)	1.6	(0.7)
Screen time	2	(4.2)	1.8	(3.7)	2.1	(4.2)	2	(4.3)	2	(4.6)
***Maternal and household variables***										
*Categorical*	*n*	*(%)*	*n*	*(%)*	*n*	*(%)*	*n*	*(%)*	*n*	*(%)*
Maternal race										
Black	611	(65)	166	(72)	156	(68)	151	(66)	123	(53)
White	273	(29)	46	(20)	61	(27)	67	(29)	94	(41)
Another race or multiple races	59	(6)	18	(8)	13	(6)	11	(5)	13	(6)
Maternal education										
<High school	52	(6)	8	(3)	15	(7)	7	(3)	20	(9)
High school degree	376	(40)	93	(40)	100	(44)	94	(41)	81	(36)
Technical school	124	(13)	30	(13)	29	(13)	38	(17)	21	(9)
College degree	237	(25)	65	(28)	53	(24)	52	(23)	61	(27)
Graduate/prof degree	147	(16)	34	(15)	28	(12)	38	(17)	45	(20)
Maternal smoking during pregnancy	81	(9)	8	(3)	25	(11)	21	(9)	22	(10)
Near road	264	(28)	83	(36)	77	(34)	55	(24)	43	(19)
*Continuous*	*mean*	*(SD)*	*mean*	*(SD)*	*mean*	*(SD)*	*mean*	*(SD)*	*mean*	*(SD)*
Adjusted HH income (USD)	17,903	(13,514)	17,129	(13,224)	17,302	(13,170)	17,932	(13,699)	19,697	(13,973)
Maternal IQ percentile	40.3	(30.7)	40.2	(29.4)	36.7	(29.8)	40.7	(31.4)	44.1	(32.4)
Maternal depression score	8.6	(7.2)	9	(8.4)	9	(7.2)	7.8	(6.3)	8.2	(6.8)
PRQ attachment score	52.9	(9.7)	53.3	(9.9)	53.2	(10.1)	52.3	(9.2)	52.5	(9.8)
***Neighborhood COI scales***	*mean*	*(SD)*	*mean*	*(SD)*	*mean*	*(SD)*	*mean*	*(SD)*	*mean*	*(SD)*
Socioeconomic	-0.113	(0.260)	-0.131	(0.271)	-0.116	(0.257)	-0.106	(0.238)	-0.101	(0.272)
Education	-0.047	(0.068)	-0.047	(0.063)	-0.052	(0.063)	-0.05	(0.064)	-0.037	(0.079)

Abbreviations: SHS = secondhand smoke, HH = household, PRQ = Parenting Relationship Questionnaire, COI = Childhood Opportunity Index

**Table 2:** CBCL scales in the analytic sample

	Full analytic sample		Sample by quartile of NDVI in 300 m							
	(*n* = 943)		Q1 (*n* = 230)		Q2 (*n* = 230)		Q3 (*n* = 229)		Q4 (*n* = 230)	
*Raw broadband scores*										
Internalizing ^a^	6.21	(6.17)	6.66	(6.82)	6.40	(6.44)	5.51	(5.05)	6.06	(6.02)
Externalizing^b^	9.18	(7.57)	9.25	(7.54)	9.25	(8.03)	9.37	(7.29)	8.56	(7.21)
*Internalizing syndrome scales* ^a^										
Emotionally reactive	1.65	(2.00)	1.76	(1.98)	1.65	(2.16)	1.42	(1.73)	1.66	(2.00)
Anxious/depressive	1.80	(2.03)	1.97	(2.27)	1.86	(2.06)	1.57	(1.72)	1.75	(2.00)
Somatic complaints	1.33	(1.75)	1.48	(1.95)	1.39	(1.74)	1.23	(1.58)	1.21	(1.61)
Withdrawn	1.43	(1.83)	1.45	(1.87)	1.50	(1.97)	1.29	(1.53)	1.44	(1.82)
*Externalizing syndrome scales* ^b^										
Attention problems	2.20	(1.94)	2.35	(1.98)	2.08	(1.92)	2.20	(1.86)	2.10	(1.95)
Aggressive behavior problems	6.98	(6.17)	6.90	(6.09)	7.17	(6.60)	7.17	(6.00)	6.46	(5.83)

^a^The range of CBCL scores in the internalizing domain in this sample was 0–44, 0–15, 0–12, 0–12, and 0–14 for the internalizing score, emotionally reactive scale, anxious/depressed scale, somatic complaints scale, and withdrawn scale, respectively

^b^The range of CBCL scores in the externalizing domain in this sample was 0–43, 0–9, and 0–36 for the externalizing score, attention problems scale, and aggressive behavior scale, respectively

The distributions of green space are shown in Table 3. Among those with a COI socioeconomic subscale value above the median versus below the median, mean (SD) of NDVI in a 300 m buffer was 0.593 (0.083) and 0.591 (0.076), respectively. Mean tree cover was 37% (SD 12%) and 38% (SD 12%) among participants with a COI socioeconomic subscale value above and below the median, respectively. In this cohort, 28% of participants lived within 300 m of the nearest park; 11% lived within 300 m of a community park (> 20 acres).

**Table 3:** Distribution of primary green space exposures in the analytic sample (*n* = 943)

Exposure	Mean	(SD)	Min	25th p.	Median	75th p.	Max
NDVI in 300 m buffer^a^	0.592	(0.08)	0.249	0.543	0.596	0.647	0.789
Tree Cover (%) ^b^	37.6	(12.0)	4.4	29.1	37.3	45.7	80.7
Park proximity (m) ^c^	795	(862)	3	282	549	987	8417

^a^ NDVI was weighted by the address history over the year prior to the age 4–6 study visit

^b^ Tree cover was defined as the percentage of census block group, weighted by address history over year prior to age 4–6 study visit

^c^ Park proximity was defined as distance from the address at the age 4–6 study visit to the edge of the nearest park

### Internalizing

**Fig. 1:**
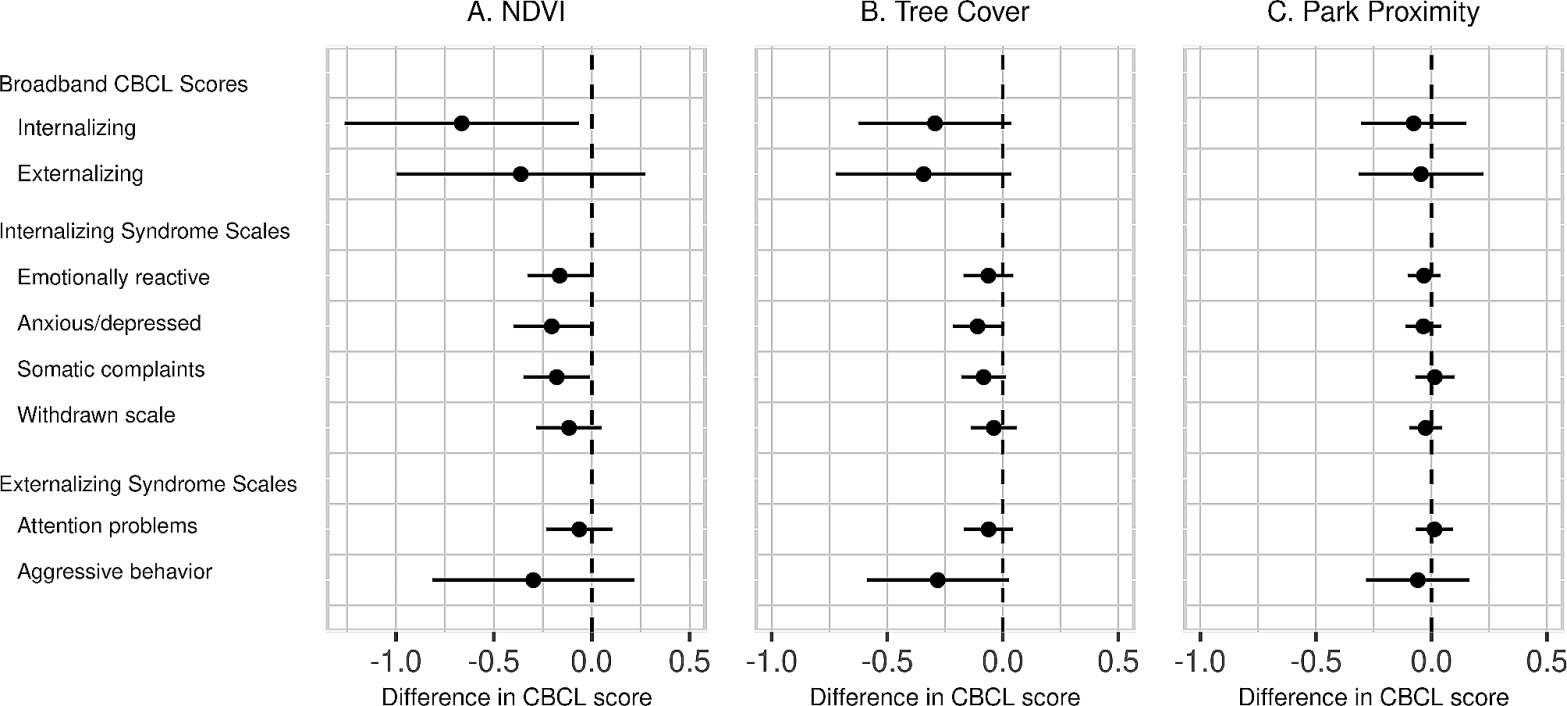
Associations between residential green space and CBCL scores. Figure legend: Differences (95% confidence intervals) in the CBCL scores are shown per (**A**) 0.1 unit higher NDVI, (**B**) 10% higher tree cover, and (**C**) 500 m further distance to the nearest park. NDVI and tree cover exposures were averaged over the residential history across the year prior to the outcome assessment. Park proximity was calculated for the current address at the time of the outcome assessment. Models were adjusted for child sex, child age at outcome assessment, maternal education, household income adjusted for household size, maternal race, socioeconomic COI scale, education COI scale, urbanicity, residential stability, maternal IQ, maternal depression, maternal smoking during pregnancy, and preterm birth

Higher levels of residential surrounding greenness were significantly associated with lower internalizing scores (Fig. 1). Effect estimates are reported per 0.1 unit NDVI, which approximates the IQR in this sample. Estimated coefficients were generally small, approximately 10% of the standard deviation of broadband scores observed in our sample. In the fully-adjusted model, a 0.1 unit higher NDVI exposure was significantly associated with a 0.66 units lower internalizing score (95% CI: -1.26, -0.07; *p* = 0.030). Higher NDVI was also significantly associated with a lower anxious/depressed score (β -0.20; 95% CI: -0.40, -0.01; *p* = 0.041) and a lower somatic complaints score (β -0.18; 95% CI: -0.35, -0.01; *p* = 0.040). Effect estimates tended to be consistent across the different covariate adjustment approaches in models 2 and 3 and in sensitivity analyses with further adjustment for living near a major roadway, child physical activity, or screen time (Supplemental Table 1). Differences in the internalizing score were similar for NDVI in larger (1000 m) buffer sizes and slightly attenuated for exposure in the 100 m buffer (Supplemental Table 2). Effect estimates were similar for exposures weighted by the address history from age 1 to 4, but were attenuated with exposures calculated at a single address representing the longest residence time (Table 4).

**Table 4:** Association of green space exposures in multiple exposure windows with child behavior

Exposure window	NDVI ^a^	Tree Cover ^a^
	β (95% CI)	β (95% CI)
Internalizing score		
Year prior to age 4–6 study visit	-0.66 (-1.26, -0.07)	-0.29 (-0.62, 0.04)
Age 1–4	-0.58 (-1.13, -0.03)	-0.31 (-0.65, 0.04)
Longest childhood address	-0.31 (-0.75, 0.13)	-0.24 (-0.53, 0.04)
Externalizing score		
Year prior to age 4–6 study visit	-0.36 (-1.00, 0.27)	-0.34 (-0.72, 0.04)
Age 1–4	-0.21 (-0.88, 0.46)	-0.31 (-0.73, 0.11)
Longest childhood address	-0.09 (-0.65, 0.48)	-0.30 (-0.68, 0.07)
Attention problems		
Year prior to age 4–6 study visit	-0.06 (-0.23, 0.10)	-0.06 (-0.17, 0.05)
Age 1–4	0.01 (-0.17, 0.19)	0.01 (-0.11, 0.12)
Longest childhood address	0.00 (-0.16, 0.16)	0.00 (-0.10, 0.10)

^a^ Difference (95% confidence interval) in CBCL scores are shown per 0.1 unit higher NDVI and per 10% higher tree cover. Models were adjusted for child sex, child age at outcome assessment, maternal education, household income adjusted for household size, maternal race, socioeconomic COI scale and education COI opportunity, urbanicity, residential stability, maternal IQ, maternal depression, PRQ attachment score, maternal smoking during pregnancy, and preterm birth

While effect estimates were in the hypothesized direction (Fig. 1), confidence intervals included the null for the association between tree cover and internalizing behavior (β -0.29 per 10% higher tree cover; 95% CI: -0.62, 0.04; *p* = 0.082). A 10% higher tree cover was associated with a 0.11 units lower anxious/depressed score (95% CI: -0.22, 0.00; *p* = 0.047). Findings were consistent across models that further adjusted for additional covariates (Supplemental Table 1) and in models with tree cover weighted by address history across various windows in early childhood (Table 4). No associations were observed between park proximity and internalizing scores in primary or sensitivity analyses (Fig. 1 and Supplemental Table 1), including those restricting the exposure metric by park size (Supplemental Table 3).

### Externalizing

All confidence intervals included the null for effect estimates of NDVI exposures on the broadband externalizing score as well as externalizing domain subscales (Fig. 1). Regression coefficients for NDVI tended to be similar across model staging adjustment (Supplemental Table 1). Estimated differences in externalizing broadband scores tended to be larger when NDVI was assessed in larger buffer sizes, but confidence intervals still included the null (Supplemental Table 2).

Estimates of associations between tree cover and each of the externalizing scales had confidence intervals that included the null (Fig. 1). In secondary analyses, we observed similarly null associations across varying exposure windows (Table 4) and additional adjustment for several covariates did not change effect estimates (Supplemental Table 1).

In primary analyses, no associations were observed with distance from the nearest park and externalizing behavior (Fig. 1). In a sensitivity analysis looking at distance to the nearest parks when classified by park size (Supplemental Table 4), there was some suggestion that living 500 m farther from the nearest community park (> 20 acres) was associated with a higher score on the attention problems scale (0.05, 95% CI: 0, 0.11, *p* = 0.07), though this result is from an exploratory sensitivity analysis and therefore should be interpreted cautiously.

### Effect modification

We assessed effect modification by the COI socioeconomic scale as a measure of neighborhood socioeconomic conditions (Table 5) and child sex (Supplemental Table 4). Analysis of effect modification by neighborhood conditions suggested a larger association between NDVI and both internalizing and externalizing scores in neighborhoods with a lower COI socioeconomic subscale value indicating lower socioeconomic opportunity. However, *p*-values for the interaction term were all greater than 0.05. No effect modification by the neighborhood socioeconomic opportunity index was observed for tree canopy or park proximity. In interaction models by child sex, estimates were larger among boys than girls for associations between NDVI and child behavior, but all *p*-values for interaction were greater than 0.05.

**Table 5:** Effect modification of associations between green space and CBCL scores by COI socioeconomic subscale

COI socioeconomic subscale percentile ^a^	NDVI ^b^ β (95% CI)	Tree Cover ^b^ β (95% CI)	Park proximity ^b^ β (95% CI)
Internalizing score			
25th p.	-0.84 (-1.76, 0.08)	-0.30 (-0.76, 0.17)	-0.20 (-0.72, 0.32)
50th p.	-0.63 (-1.19, -0.08)	-0.29 (-0.62, 0.03)	-0.13 (-0.44, 0.19)
75th p.	-0.40 (-1.04, 0.24)	-0.29 (-0.66, 0.08)	-0.04 (-0.27, 0.19)
Interaction *p*-value	0.46	0.98	0.56
Externalizing score			
25th p.	-0.63 (-1.51, 0.25)	-0.34 (-0.83, 0.15)	-0.29 (-0.87, 0.30)
50th p.	-0.32 (-0.93, 0.30)	-0.34 (-0.72, 0.04)	-0.14 (-0.50, 0.22)
75th p.	0.04 (-0.73, 0.81)	-0.35 (-0.83, 0.14)	0.02 (-0.26, 0.30)
Interaction *p*-value	0.25	0.98	0.31
Attention problems			
25th p.	-0.11 (-0.31, 0.09)	-0.05 (-0.19, 0.08)	-0.03 (-0.18, 0.12)
50th p.	-0.06 (-0.23, 0.11)	-0.06 (-0.17, 0.04)	0.00 (-0.10, 0.09)
75th p.	0.00 (-0.23, 0.24)	-0.07 (-0.20, 0.06)	0.02 (-0.06, 0.11)
Interaction *p*-value	0.40	0.86	0.50

^a^ Effect modification was assessed by including a multiplicative interaction term between green space and the COI socioeconomic scale in the model. A higher value in the neighborhood scale indicates more socioeconomic opportunity. *P*-values shown are for the interaction term. Models were adjusted for child sex, child age at outcome assessment, maternal education, household income adjusted for household size, maternal race, socioeconomic COI scale and education COI opportunity, urbanicity, residential stability, maternal IQ, maternal depression, PRQ attachment score, maternal smoking during pregnancy, and preterm birth

^b^ Differences in CBCL scores (95% confidence intervals) are shown for a 0.1 unit higher NDVI, 10% higher tree cover, and living 500 m closer to a park, at the 25th percentile, 50th percentile, and 75th percentile of the COI socioeconomic scale

## Discussion

In this study of over 900 children aged 4–6 years enrolled in a longitudinal birth cohort, higher total residential surrounding greenness within 300 m of the residential location, but not tree cover or park proximity, was associated with lower levels of internalizing problems in young children ages 4–6. Results in secondary analyses suggest that these associations were driven largely by items on the anxious/depressed and somatic complaints syndrome scales. No associations were observed between green space and externalizing outcomes. Results from sensitivity analyses were generally consistent with conclusions from the primary analysis.

Our study further supports and extends existing literature suggesting a relationship between green space and internalizing behaviors among school-age children. For example, Madzia et al. observed lower anxiety t-scores with higher NDVI in 800 m, as well as lower depression and somatization t-scores with higher NDVI in 200 m [16]. These associations were identified among children at age 12 years, but not at age 7 years, using the Behavior Assessment System for Children, Second Edition (BASC-2) to assess behavior in a cohort in Cincinnati, OH. Amoly et al. similarly identified associations between higher green space and lower internalizing behaviors in a pediatric cohort ages 7–10, using the Strengths and Difficulties Questionnaire (SDQ) to assess child behaviors [44]. Other studies provide more limited evidence for associations between green space and internalizing behavior, with some associations only observed when accounting for exposure at the school location, only with exposure in larger buffer sizes, only among some subgroups, or among adolescents [15, 45–49]. Our study addresses some limitations identified in this prior work, including adjusting for factors that may have biased previously observed associations away from the null such as maternal depression. We also contribute to this literature by investigating associations earlier in the life course, and our results suggest the relevance of green space in this early window for internalizing behavior outcomes. Furthermore, to reduce exposure misclassification we obtained detailed address histories and used NDVI exposures at a fine spatial scale, which allowed us to investigate exposures averaged in multiple buffer sizes and across multiple periods in early childhood.

In contrast to much of the prior research, we did not observe an association between green space and externalizing behavior. Studies relying on parent-report of child behaviors include those that identified associations between higher neighborhood greenness and lower scores on broadband externalizing measures, including among nationally representative samples of children in Australia and South Korea [48–50]. Among those that further probed associations with subsets of behaviors within the externalizing domain, several have identified relationships with attention problems and hyperactivity scores [44, 47, 49, 51]. Some studies have also identified associations between more green space and lower conduct problem or aggressive behavior scores in older children and adolescents [16, 47, 49, 52]. There is variability in the published literature on this topic; several studies have reported effects only in some subgroups or null findings across all externalizing scales [45, 50, 53]. Though some of our sensitivity analyses hinted at associations with attention problems or aggressive behavior, we conducted many tests without adjusting for multiple comparisons and thus these sensitivity results should be interpreted cautiously.

Many behaviors within the externalizing domain, including those related to ADHD, may be more likely to be identified as problems once children reach school-age; therefore, associations between green space and externalizing behaviors may be harder to detect among younger children. Some of the strongest observational evidence for the relationship between green space and attention-related outcomes comes from several large population-based cohort studies from urban settings in multiple countries, that suggest an inverse association between NDVI and ADHD diagnosis that may be explained in part by reduced exposure to traffic-related air pollution [11, 54–56]. Notably, our study included a younger sample than these prior studies, which included children ranging in age from 7 to 17 years.

While an array of potential benefits of tree canopy have been hypothesized, the quantitative evidence for the relationship of tree cover with child health is still limited [57]. We did not observe associations of either internalizing or externalizing behaviors with tree canopy in the current study, though effect estimates were in the hypothesized direction. One limitation of the tree cover exposure measure we used was that it was only available at the census block group geography. This limited our ability to determine whether estimates differed from NDVI due to the presence of tree cover or due to the choice of areal unit (300 m radial buffer versus census block group) and prevented us from exploring associations with tree canopy across multiple buffer sizes. The location of the trees may also be relevant; our exposure measure did not distinguish between trees lining the roadway versus aggregated in park areas. More research is needed to evaluate these questions.

Health promoting associations have also been observed when access to green space was operationalized as proximity to the nearest urban green space or city park [58, 59]. Unlike these previous studies, we did not observe associations between proximity from parks and any subscales. One limitation of the current study is that we do not have a measure of neighborhood park use or whether the nearest park includes a playground that may be more often frequented by younger children and their caregivers. In contrast, the NDVI measure represents a cumulative exposure that reflects both intentional and unintentional green space exposure, which may in part explain why we observed associations of internalizing behavior with NDVI but not with park proximity. Perceptions of safety and availability of park amenities for families with young children including shaded areas, may also influence park use and were not captured in our exposure measure.

Multiple mechanisms have been theorized for the relationship between green space and health outcomes, including child behavior [57, 60, 61]. Experimental evidence suggests that cognitive processing tasks requiring control of attention towards both internal and external stimuli or suppression of both internal and external distractors may be sensitive to features of natural environments [21]. Our finding of a relationship of internalizing behavior with overall greenness, but not park proximity, is consistent with these hypothesized mechanisms. Some studies also suggest that green space may reduce exposure to air pollutants, though this literature lacks formal mediation analyses [60]. In our analysis, the results did not substantively change when distance to the nearest major roadway was added to the models as a proxy for traffic-related air pollution. Across all ages, urban green space may also provide spaces for physical activity, which may in turn improve both executive functioning and mental health [62]. In our models, further adjustment for child physical activity did not change the results though formal investigation of mediation in future work is warranted, ideally with objective physical activity measurement.

Green space may be particularly important for children and families with access to fewer resources. Populations with lower SES are more likely to experience higher levels of adverse stressors and environmental exposures and may be more reliant on resources within the residential neighborhood, each of which may suggest a greater potential benefit of residential green space exposures [63]. Several studies have identified relationships between green space and child behavioral and mental health that are more pronounced among those with lower parent educational attainment levels, lower household income, or lower area-level SES [45, 53, 59, 64]. However, others have identified stronger associations between green space and health in higher income areas and suggest that this may be due to higher perceived quality of green space translating to increased use of those spaces and improved health outcomes [46, 65]. Overall, evidence of effect modification by SES at either individual or neighborhood levels in prior studies is mixed, with some estimating a stronger association with green space in the group with lower SES and others estimating a stronger association with green space in the group with higher SES [45, 46, 53, 59, 65]. Though not statistically significant, the magnitude of the association between NDVI and internalizing behavior in this study was estimated to be larger for those in neighborhoods with lower socioeconomic opportunity. Further confirmation of this trend in future work could support urban green space as a resource to promote health equity.

This analysis from a well-characterized, socioeconomically and racially diverse pediatric cohort in an urban area in the US contributes to the growing literature on the relationship between green space and mental health and addresses several current gaps in our knowledge of the relationship of green spaces to children’s development. Few studies have explored relationships of natural environments to child behavior in the US in a cohort of this size and fewer still have examined multiple forms of green space. Importantly, we were able to examine multiple green space exposures, including a measure of green structure (i.e. tree cover) as well as public green space (i.e. park proximity), using a highly-resolved address history across childhood. An additional strength of this study was our ability to account for a suite of potential confounders at both the individual and neighborhood levels.

This analysis was also subject to several limitations. First, while the CBCL is a well-established, validated, widely-used tool in research settings and appropriate for this age, it relies on parent report of child behaviors and may be subject to outcome misclassification. Some mothers may be more or less likely to report problems to clinicians in a research setting or responses may be relative to other children the parent observes in the child’s peer group, which may have on average either more or fewer problems than national norms. Underreporting of behavior may have contributed to attenuation of any true effects in this analysis. Second, due to the particular urban setting in which the CANDLE cohort resides in Shelby County, green space measures were generally high. It is possible that any effects of green space on externalizing outcomes may occur at lower levels and taper off at higher levels of exposure, which could explain the null results observed here for externalizing behavior and attention problems. Lastly, we did not have information about green space at any childcare locations or the amount of time children spent outside in natural environments. While residential green space is frequently considered in the literature, these measures may not fully capture the relevant park spaces or tree canopy exposures for children.

## Conclusions

Our finding of a relationship between surrounding greenness and internalizing score extends the literature in older samples to this younger age group, highlighting the importance of this relationship early in the life course. Future work using data from school-age and adolescence in the CANDLE cohort may be able to examine this question with more refined exposure and outcome assessments, including incorporation of school-based exposures and child-report of externalizing and internalizing symptoms.

## Electronic supplementary material

Below is the link to the electronic supplementary material.

Supplementary Material 1

## Data Availability

The data utilized for this study are not publicly available but de-identified data may be available on request, subject to approval by the internal review board and under a formal data use agreement. Contact the corresponding author for more information.

## References

[1] Minh A, Muhajarine N, Janus M, Brownell M, Guhn M. A review of neighborhood effects and early child development: how, where, and for whom, do neighborhoods matter? Health Place. 2017;46:155–74. 28528276 10.1016/j.healthplace.2017.04.012

[2] Humphrey JL, Root ED. Spatio-temporal neighborhood impacts on internalizing and externalizing behaviors in U.S. elementary school children: effect modification by child and family socio-demographics. Soc Sci Med. 2017;180:52–61. 28324791 10.1016/j.socscimed.2017.03.014

[3] Leventhal T, Brooks-Gunn J. Moving to opportunity: an experimental study of Neighborhood effects on Mental Health. Am J Public Health. 2003;93(9):1576–82. 12948983 10.2105/AJPH.93.9.1576 PMC1448013

[4] Fyfe-Johnson AL, Hazlehurst MF, Perrins SP, Bratman GN, Thomas R, Garrett KA, et al. Nat Children’s Health: Syst Rev Pediatr. 2021;148(4):e2020049155. 10.1542/peds.2020-049155 34588297

[5] Jimenez MP, Aris IM, Rifas-Shiman S, Young J, Tiemeier H, Hivert MF, et al. Early life exposure to greenness and executive function and behavior: an application of inverse probability weighting of marginal structural models. Environ Pollut. 2021;291:118208. 34740291 10.1016/j.envpol.2021.118208 PMC9208930

[6] Jimenez MP, Shoaff J, Kioumourtzoglou MA, Korrick S, Rifas-Shiman SL, Hivert MF, et al. Early-life exposure to Green Space and Mid-childhood Cognition in the project viva cohort, Massachusetts. Am J Epidemiol. 2022;191(1):115–25. 34308473 10.1093/aje/kwab209 PMC8897997

[7] Achenbach TM, Ivanova MY, Rescorla LA, Turner LV, Althoff RR. Internalizing/Externalizing problems: review and recommendations for clinical and Research Applications. J Am Acad Child Adolesc Psychiatry. 2016;55(8):647–56. 27453078 10.1016/j.jaac.2016.05.012

[8] Erskine HE, Norman RE, Ferrari AJ, Chan GCK, Copeland WE, Whiteford HA, et al. Long-term outcomes of Attention-Deficit/Hyperactivity disorder and Conduct Disorder: a systematic review and Meta-analysis. J Am Acad Child Adolesc Psychiatry. 2016;55(10):841–50. 27663939 10.1016/j.jaac.2016.06.016

[9] Liu J, Chen X, Lewis G. Childhood internalizing behaviour: analysis and implications: Childhood internalizing behaviour. J Psychiatr Ment Health Nurs. 2011;18(10):884–94. 22070805 10.1111/j.1365-2850.2011.01743.x PMC5675073

[10] Zare Sakhvidi MJ, Knobel P, Bauwelinck M, de Keijzer C, Boll LM, Spano G, et al. Greenspace exposure and children behavior: a systematic review. Sci Total Environ. 2022;824:153608. 35134416 10.1016/j.scitotenv.2022.153608

[11] Thygesen M, Engemann K, Holst GJ, Hansen B, Geels C, Brandt J, et al. The Association between Residential Green Space in Childhood and Development of attention deficit hyperactivity disorder: a Population-based Cohort Study. Environ Health Perspect. 2020;128(12):127011. 33351671 10.1289/EHP6729 PMC7755168

[12] Dadvand P, Nieuwenhuijsen MJ, Esnaola M, Forns J, Basagaña X, Alvarez-Pedrerol M, et al. Green spaces and cognitive development in primary schoolchildren. PNAS. 2015;112(26):7937–42. 26080420 10.1073/pnas.1503402112 PMC4491800

[13] Dadvand P, Tischer C, Estarlich M, Llop S, Dalmau-Bueno A, López-Vicente M, et al. Lifelong residential exposure to Green Space and attention: a Population-based prospective study. Environ Health Perspect. 2017;125(9):097016. 28934095 10.1289/EHP694 PMC5915181

[14] Bezold CP, Banay RF, Coull BA, Hart JE, James P, Kubzansky LD, et al. The relationship between surrounding greenness in childhood and adolescence and depressive symptoms in adolescence and early adulthood. Ann Epidemiol. 2018;28(4):213–9. 29426730 10.1016/j.annepidem.2018.01.009 PMC5869153

[15] Hartley K, Perazzo J, Brokamp C, Gillespie GL, Cecil KM, LeMasters G, et al. Residential surrounding greenness and self-reported symptoms of anxiety and depression in adolescents. Environ Res. 2021;194:110628. 33345894 10.1016/j.envres.2020.110628 PMC9933414

[16] Madzia J, Ryan P, Yolton K, Percy Z, Newman N, LeMasters G, et al. Residential Greenspace Association with Childhood behavioral outcomes. J Pediatr. 2019;207:233–40. 30545565 10.1016/j.jpeds.2018.10.061 PMC6440820

[17] Nordbø ECA. Neighborhood green spaces, facilities and population density as predictors of activity participation among 8-year-olds: a cross-sectional GIS study based on the Norwegian mother and child cohort study. 2019;22. 10.1186/s12889-019-7795-9 PMC6822450 31666049

[18] Jennings V, Bamkole O. The relationship between Social Cohesion and Urban Green Space: An Avenue for Health Promotion. IJERPH. 2019;16(3):452. 30720732 10.3390/ijerph16030452 PMC6388234

[19] Kaplan S, Berman MG. Directed attention as a common resource for executive functioning and self-regulation. Perspect Psychol Sci. 2010;5(1):43–57. 26162062 10.1177/1745691609356784

[20] Ohly H, White MP, Wheeler BW, Bethel A, Ukoumunne OC, Nikolaou V, et al. Attention restoration theory: a systematic review of the attention restoration potential of exposure to natural environments. J Toxicol Environ Health Part B. 2016;19(7):305–43. 10.1080/10937404.2016.1196155 27668460

[21] Stevenson MP, Schilhab T, Bentsen P. Attention restoration theory II: a systematic review to clarify attention processes affected by exposure to natural environments. J Toxicol Environ Health Part B. 2018;21(4):227–68. 10.1080/10937404.2018.1505571 30130463

[22] Ulrich RS, Simons RF, Losito BD, Fiorito E, Miles MA, Zelson M. Stress recovery during exposure to natural and urban environments. J Environ Psychol. 1991;11(3):201–30. 10.1016/S0272-4944(05)80184-7

[23] Labib SM, Lindley S, Huck JJ. Spatial dimensions of the influence of urban green-blue spaces on human health: a systematic review. Environ Res. 2020;180:108869. 31722804 10.1016/j.envres.2019.108869

[24] Gascon M, Triguero-Mas M, Martínez D, Dadvand P, Forns J, Plasència A, et al. Mental Health benefits of long-term exposure to Residential Green and Blue spaces: a systematic review. IJERPH. 2015;12(4):4354–79. 25913182 10.3390/ijerph120404354 PMC4410252

[25] Palmer FB, Anand KJS, Graff JC, Murphy LE, Qu Y, Völgyi E, et al. Early Adversity, Socioemotional Development, and stress in Urban 1-Year-old children. J Pediatr. 2013;163(6):1733–1739e1. 24070827 10.1016/j.jpeds.2013.08.030

[26] Völgyi E, Carroll K, Hare M, Ringwald-Smith K, Piyathilake C, Yoo W, et al. Dietary patterns in pregnancy and effects on Nutrient Intake in the Mid-south: the conditions affecting Neurocognitive Development and Learning in Early Childhood (CANDLE) study. Nutrients. 2013;5(5):1511–30. 23645026 10.3390/nu5051511 PMC3708333

[27] ECHO-PATHWAYS [Internet]. Available from: https://deohs.washington.edu/echo/.

[28] Achenbach TM, Rescorla LA. Manual for the ASEBA Preschool forms & profiles. Burlington, VT: University of Vermont, Research Center for Children, Youth, & Families; 2000.

[29] Hudziak JJ, Copeland W, Stanger C, Wadsworth M. Screening for DSM-IV externalizing disorders with the child Behavior Checklist: a receiver-operating characteristic analysis. J Child Psychol & Psychiat. 2004;45(7):1299–307. 15335349 10.1111/j.1469-7610.2004.00314.x

[30] Roy D, Zhang H. NASA Global Web-Enabled Landsat Data Annual Global 30 m V031 [Data set]. NASA EOSDIS Land Processes DAAC. 2019 [cited 2020 Oct 15]. GWELD. 10.5067/MEaSUREs/GWELD/GWELDYR.031.

[31] van den Annerstedt M, Mudu P, Uscila V, Barrdahl M, Kulinkina A, Staatsen B, et al. Development of an urban green space indicator and the public health rationale. Scand J Public Health. 2016;44(2):159–67. 26573907 10.1177/1403494815615444

[32] Smith G, Cirach M, Swart W, Dėdelė A, Gidlow C, van Kempen E, et al. Characterisation of the natural environment: quantitative indicators across Europe. Int J Health Geogr. 2017;16(1):16. 28446187 10.1186/s12942-017-0090-z PMC5406880

[33] United States Environmental Protection Agency. EnviroAtlas. Community Metrics: Percent Tree Cover. [cited 2020 Nov 9]. EnviroAtlas. Available from: enviroatlas.epa.gov/enviroatlas.

[34] The Trust for Public Land. ParkServe Data Downloads. [cited 2020 Mar 10]. ParkServe. Available from: www.tpl.org/parkserve/downloads.

[35] Cohen DA, Han B, Nagel CJ, Harnik P, McKenzie TL, Evenson KR, et al. The First National Study of Neighborhood Parks. Am J Prev Med. 2016;51(4):419–26. 27209496 10.1016/j.amepre.2016.03.021 PMC5030121

[36] Armour P, Burkhauser R, Larrimore J. Using the Pareto Distribution to Improve Estimates of Topcoded Earnings. [Internet]. 2014 [cited 2021 Aug 4]. Available from: www.census.gov/ces.

[37] What are Equivalence Scales? OECD Project on Income Distribution and Poverty [Internet]. Available from: https://www.oecd.org/social/inequality.htm.

[38] Wechsler D. Wechsler abbreviated scale of intelligence. San Antonio, TX: Psychological Corporation; 1999.

[39] McCrimmon A, Smith A. Review of the Wechsler Abbreviated Scale of Intelligence, Second Edition (WASI-II). J Psychoeducational Assess. 2013;31(3):337–41. 10.1177/0734282912467756

[40] Radloff L. The CES-D scale: a self report depression scale for research in the general population. Appl Psychol Measurements. 1977;1:385–401. 10.1177/014662167700100306

[41] Kamphaus R, Reynolds C. Parenting Relationship Questionnaire (PRQ). APA PsychTests. 2006.

[42] Noelke C, McArdle N, Baek M, Huntington N, Huber R, Hardy E et al. Childhood Opportunity Index 2.0 Technical Documentation [Internet]. 2020. Available from: diversitydatakids.org/research-library/research-brief/how-we-built-it. 10.1377/hlthaff.2020.00735 33017244

[43] Sontag-Padilla L, Burns R, Shih R, Griffin B, Martin L, Chandra A et al. The Urban Child Institute CANDLE Study: Methodological Overview and Baseline Sample Description [Internet]. RAND Corporation; 2015 [cited 2021 Apr 24]. Available from: http://www.rand.org/pubs/research_reports/RR1336.html.

[44] Amoly E, Dadvand P, Forns J, López-Vicente M, Basagaña X, Julvez J, et al. Green and blue spaces and behavioral development in Barcelona Schoolchildren: the BREATHE Project. Environ Health Perspect. 2014;122(12):1351–8. 25204008 10.1289/ehp.1408215 PMC4256702

[45] Richardson EA, Pearce J, Shortt NK, Mitchell R. The role of public and private natural space in children’s social, emotional and behavioural development in Scotland: a longitudinal study. Environ Res. 2017;158:729–36. 28750342 10.1016/j.envres.2017.07.038 PMC5571194

[46] McEachan RRC, Yang TC, Roberts H, Pickett KE, Arseneau-Powell D, Gidlow CJ, et al. Availability, use of, and satisfaction with green space, and children’s mental wellbeing at age 4 years in a multicultural, deprived, urban area: results from the born in Bradford cohort study. Lancet Planet Health. 2018;2(6):e244–54. 29880156 10.1016/S2542-5196(18)30119-0

[47] Liao J, Yang S, Xia W, Peng A, Zhao J, Li Y, et al. Associations of exposure to green space with problem behaviours in preschool-aged children. Int J Epidemiol. 2020;49(3):944–53. 31782776 10.1093/ije/dyz243

[48] Bijnens EM, Derom C, Thiery E, Weyers S, Nawrot TS. Residential green space and child intelligence and behavior across urban, suburban, and rural areas in Belgium: a longitudinal birth cohort study of twins. Markevych I, editor. PLoS Med. 2020;17(8):e1003213. 32810193 10.1371/journal.pmed.1003213 PMC7446904

[49] Lee M, Kim S, Ha M. Community greenness and neurobehavioral health in children and adolescents. Sci Total Environ. 2019;672:381–8. 30959304 10.1016/j.scitotenv.2019.03.454

[50] Feng X, Astell-Burt T. Residential Green Space Quantity and Quality and Child Well-being: a longitudinal study. Am J Prev Med. 2017;53(5):616–24. 28864128 10.1016/j.amepre.2017.06.035

[51] Andrusaityte S, Grazuleviciene R, Dedele A, Balseviciene B. The effect of residential greenness and city park visiting habits on preschool children’s mental and general health in Lithuania: a cross-sectional study. Int J Hyg Environ Health. 2020;223(1):142–50. 31564508 10.1016/j.ijheh.2019.09.009

[52] Younan D, Tuvblad C, Li L, Wu J, Lurmann F, Franklin M, et al. Environmental determinants of aggression in adolescents: role of Urban Neighborhood Greenspace. J Am Acad Child Adolesc Psychiatry. 2016;55(7):591–601. 27343886 10.1016/j.jaac.2016.05.002 PMC4924128

[53] Flouri E, Midouhas E, Joshi H. The role of urban neighbourhood green space in children’s emotional and behavioural resilience. J Environ Psychol. 2014;40:179–86. 10.1016/j.jenvp.2014.06.007

[54] Markevych I, Tesch F, Datzmann T, Romanos M, Schmitt J, Heinrich J. Outdoor air pollution, greenspace, and incidence of ADHD: a semi-individual study. Sci Total Environ. 2018;642:1362–8. 30045516 10.1016/j.scitotenv.2018.06.167

[55] Yang BY, Zeng XW, Markevych I, Bloom MS, Heinrich J, Knibbs LD, et al. Association between Greenness Surrounding Schools and kindergartens and Attention-Deficit/Hyperactivity disorder in children in China. JAMA Netw Open. 2019;2(12):e1917862. 31851349 10.1001/jamanetworkopen.2019.17862 PMC6991306

[56] Donovan GH, Michael YL, Gatziolis D, Mannetje A, ’t, Douwes J. Association between exposure to the natural environment, rurality, and attention-deficit hyperactivity disorder in children in New Zealand: a linkage study. Lancet Planet Health. 2019;3(5):e226–34. 31128768 10.1016/S2542-5196(19)30070-1

[57] Wolf KL, Lam ST, McKeen JK, Richardson GRA, van den Bosch M, Bardekjian AC. Urban Trees and Human Health: A Scoping Review IJERPH. 2020;17(12):4371. 10.3390/ijerph17124371 PMC7345658 32570770

[58] Markevych I, Tiesler CMT, Fuertes E, Romanos M, Dadvand P, Nieuwenhuijsen MJ, et al. Access to urban green spaces and behavioural problems in children: results from the GINIplus and LISAplus studies. Environ Int. 2014;71:29–35. 24953038 10.1016/j.envint.2014.06.002

[59] Balseviciene B, Sinkariova L, Grazuleviciene R, Andrusaityte S, Uzdanaviciute I, Dedele A, et al. Impact of residential greenness on Preschool Children’s emotional and behavioral problems. IJERPH. 2014;11(7):6757–70. 24978880 10.3390/ijerph110706757 PMC4113842

[60] Markevych I, Schoierer J, Hartig T, Chudnovsky A, Hystad P, Dzhambov AM, et al. Exploring pathways linking greenspace to health: theoretical and methodological guidance. Environ Res. 2017;158:301–17. 28672128 10.1016/j.envres.2017.06.028

[61] Mygind L, Kurtzhals M, Nowell C, Melby PS, Stevenson MP, Nieuwenhuijsen M, et al. Landscapes of becoming social: a systematic review of evidence for associations and pathways between interactions with nature and socioemotional development in children. Environ Int. 2021;146:106238. 33189991 10.1016/j.envint.2020.106238

[62] Christiansen L, Beck MM, Bilenberg N, Wienecke J, Astrup A, Lundbye-Jensen J. Effects of Exercise on Cognitive performance in children and adolescents with ADHD: potential mechanisms and evidence-based recommendations. JCM. 2019;8(6):841. 31212854 10.3390/jcm8060841 PMC6617109

[63] Rigolon A, Browning MHEM, McAnirlin O, Yoon H. (Violet). Green Space and Health Equity: a systematic review on the potential of Green Space to Reduce Health disparities. IJERPH. 2021;18(5):2563. 33806546 10.3390/ijerph18052563 PMC7967323

[64] Pérez-del-Pulgar C, Anguelovski I, Cole HVS, de Bont J, Connolly J, Baró F, et al. The relationship between residential proximity to outdoor play spaces and children’s mental and behavioral health: the importance of neighborhood socio-economic characteristics. Environ Res. 2021;200:111326. 34029548 10.1016/j.envres.2021.111326

[65] Feng X, Astell-Burt T. The relationship between Neighbourhood Green Space and Child Mental Wellbeing depends upon whom you ask: Multilevel evidence from 3083 children aged 12–13 years. IJERPH. 2017;14(3):235. 28264461 10.3390/ijerph14030235 PMC5369071

