# Correction: Associations of residential green space with internalizing and externalizing behavior in early childhood

**DOI:** 10.1186/s12940-024-01112-z

**Published:** 2024-09-12

**Authors:** Marnie F. Hazlehurst, Anjum Hajat, Pooja S. Tandon, Adam A. Szpiro, Joel D. Kaufman, Frances A. Tylavsky, Marion E. Hare, Sheela Sathyanarayana, Christine T. Loftus, Kaja Z. LeWinn, Nicole R. Bush, Catherine J. Karr

**Affiliations:** 1 Department of Epidemiology, Department of Environmental & Occupational Health Sciences, University of Washington School of Public Health, 4225 Roosevelt Way NE, Seattle, WA 98105 USA; 2 Department of Epidemiology, University of Washington School of Public Health, Seattle, WA USA; 3 Seattle Children’s Research Institute, Department of Pediatrics, University of Washington School of Medicine, Seattle, WA USA; 4 Department of Biostatistics, University of Washington School of Public Health, Seattle, WA USA; 5 Department of Environmental & Occupational Health Sciences, Department of Epidemiology, Division of General Internal Medicine, Department of Medicine, University of Washington School of Public Health, University of Washington School of Medicine, Seattle, WA USA; 6 Department of Preventive Medicine, University of Tennessee Health Science Center, Memphis, TN USA; 7 Seattle Children’s Research Institute; Department of Pediatrics, University of Washington School of Medicine; Department of Environmental & Occupational Health Sciences, University of Washington School of Public Health, Seattle, WA USA; 8 Department of Environmental & Occupational Health Sciences, University of Washington School of Public Health, Seattle, WA USA; 9 Department of Psychiatry School of Medicine, University of California San Francisco, San Francisco, CA USA; 10 Department of Psychiatry, Department of Pediatrics, School of Medicine, University of California San Francisco, San Francisco, CA USA; 11 Department of Pediatrics, Department of Environmental & Occupational Health Sciences, University of Washington School of Medicine, University of Washington School of Public Health, Seattle, WA USA

**Correction: Environ Health 23, 17 (2024)**

10.1186/s12940-024-01051-9

Following publication of the original article [1], the authors identified an error in Table 5. In the PDF version, Table 5, there were missing data.

The correct table is provided below and the original article has been updated.

**Table 1:** 

COI socioeconomic subscale percentile ^a^	NDVI ^b^ β (95% CI)	Tree Cover ^b^ β (95% CI)	Park proximity ^b^ β (95% CI)
Internalizing score			
25th p.	-0.84 (-1.76, 0.08)	-0.30 (-0.76, 0.17)	-0.20 (-0.72, 0.32)
50th p.	-0.63 (-1.19, -0.08)	-0.29 (-0.62, 0.03)	-0.13 (-0.44, 0.19)
75th p.	-0.40 (-1.04, 0.24)	-0.29 (-0.66, 0.08)	-0.04 (-0.27, 0.19)
Interaction p-value	0.46	0.98	0.56
Externalizing score			
25th p.	-0.63 (-1.51, 0.25)	-0.34 (-0.83, 0.15)	-0.29 (-0.87, 0.30)
50th p.	-0.32 (-0.93, 0.30)	-0.34 (-0.72, 0.04)	-0.14 (-0.50, 0.22)
75th p.	0.04 (-0.73, 0.81)	-0.35 (-0.83, 0.14)	0.02 (-0.26, 0.30)
Interaction p-value	0.25	0.98	0.31
Attention problems			
25th p.	-0.11 (-0.31, 0.09)	-0.05 (-0.19, 0.08)	-0.03 (-0.18, 0.12)
50th p.	-0.06 (-0.23, 0.11)	-0.06 (-0.17, 0.04)	0.00 (-0.10, 0.09)
75th p.	0.00 (-0.23, 0.24)	-0.07 (-0.20, 0.06)	0.02 (-0.06, 0.11)
Interaction p-value	0.40	0.86	0.50
