# Supplementary material for: Associations of residential green space with internalizing and externalizing behavior in early childhood
Source: Environ Health. 2024 Feb 8;23:17. doi: 10.1186/s12940-024-01051-9 (PMC10851463; doi:10.1186/s12940-024-01051-9)
Supplement: Supplementary file 1 — Supplementary Material 1 [file 12940_2024_1051_MOESM1_ESM.docx]

**Supplementary Materials**

**Associations of residential green space with internalizing and externalizing behavior in early childhood**

Marnie F. Hazlehurst, Anjum Hajat, Pooja S. Tandon, Adam A. Szpiro, Joel D. Kaufman, Frances A. Tylavsky, Marion E. Hare, Sheela Sathyanarayana, Christine T. Loftus, Kaja Z. LeWinn, Nicole R. Bush, Catherine J. Karr.

**Contents**

**Supplemental Figure 1**. Flowchart of inclusion in the analytic sample for each analysis.

**Supplemental Table 1**. Associations between residential green space and child behavior in models with varying covariate adjustment.

**Supplemental Table 2**. Associations with NDVI in multiple buffer sizes.

**Supplemental Table 3**. Associations between distance to the nearest small, neighborhood, and community park and child behavior.

**Supplemental Table 4**. Association between green space and child CBCL scores among boys and girls.

**Supplemental** **Figure 1.** Flowchart of inclusion in the analytic sample for each analysis.


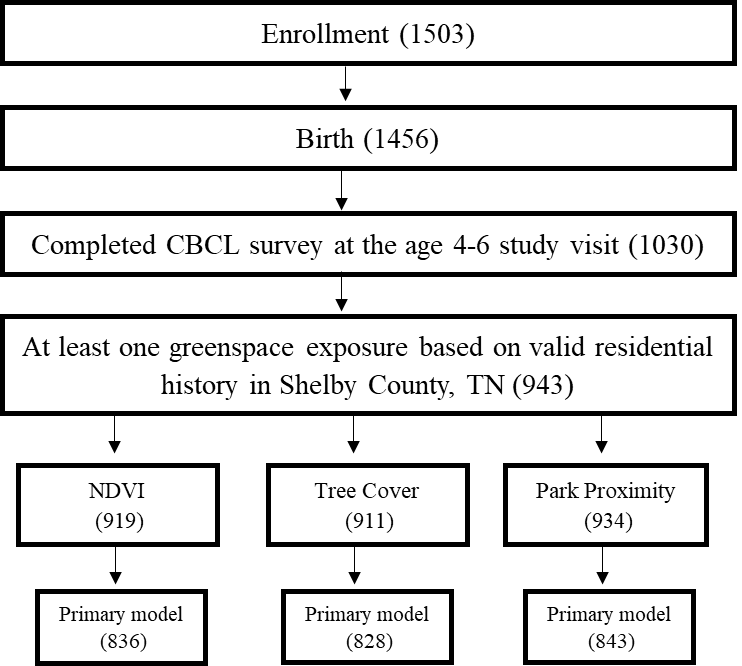


Between enrollment and birth, 47 mothers were lost to follow-up. At the age 4-6 follow-up visit, the outcomes were ascertained for 71% of the children in the cohort at birth. Addresses used to assign greenspace exposures in this study were considered invalid for the purposes of this analysis if they could not be geocoded to a more granular level than the zip code or fell outside of the boundary of Shelby County, TN. The primary exposures in this analysis required a valid address history over the year prior to the age 4-6 visit (for NDVI and tree canopy) or a valid address at the time of the visit (for park proximity). When averaging exposures across the year prior to the study visit, exposures were excluded if more than 25% of the relevant residential history was missing, whereas park proximity measures only required an address on the date of the study visit resulting in some participants being assigned a park proximity measure but not NDVI or tree cover exposures. Tree canopy data from EnviroAtlas were not available for a small number of block groups within Shelby County; participants within those block groups (n=8) were assigned NDVI exposures but not tree cover*.* Reductions in sample size in the primary models were primarily due to missing household income data or missing maternal depression.

**Supplemental** **Table 1.** Associations between residential green space and child behavior in models with varying covariate adjustment.

| **Covariate adjustment** ^a^ | **NDVI**  β (95% CI) | **Tree Canopy**  β (95% CI) | **Park Proximity**  β (95% CI) |
| --- | --- | --- | --- |
| Internalizing score |  |  |  |
| Model 1 | -0.49 (-1.09, 0.11) | -0.15 (-0.51, 0.20) | -0.08 (-0.28, 0.11) |
| Model 2 | -0.59 (-1.22, 0.03) | -0.15 (-0.50, 0.19) | -0.04 (-0.27, 0.18) |
| Model 3 | -0.66 (-1.26, -0.07) | -0.29 (-0.62, 0.04) | -0.08 (-0.31, 0.15) |
| Adjusted for near-road | -0.70 (-1.32, -0.08) | -0.30 (-0.63, 0.03) | -0.08 (-0.31, 0.15) |
| Adjusted for physical activity | -0.67 (-1.27, -0.06) | -0.28 (-0.61, 0.05) | -0.09 (-0.32, 0.14) |
| Adjusted for screen time | -0.64 (-1.25, -0.04) | -0.28 (-0.62, 0.05) | -0.08 (-0.30, 0.15) |
| Externalizing domain score |  |  |  |
| Model 1 | -0.26 (-0.89, 0.36) | -0.18 (-0.57, 0.21) | -0.04 (-0.27, 0.19) |
| Model 2 | -0.31 (-0.96, 0.34) | -0.21 (-0.59, 0.18) | -0.01 (-0.28, 0.26) |
| Model 3 | -0.36 (-1.00, 0.27) | -0.34 (-0.72, 0.04) | -0.05 (-0.32, 0.23) |
| Adjusted for near-road | -0.36 (-1.01, 0.29) | -0.34 (-0.72, 0.04) | -0.04 (-0.31, 0.22) |
| Adjusted for physical activity | -0.37 (-1.01, 0.27) | -0.32 (-0.71, 0.06) | -0.06 (-0.33, 0.21) |
| Adjusted for screen time | -0.33 (-0.97, 0.31) | -0.34 (-0.72, 0.04) | -0.05 (-0.32, 0.22) |
| Attention problems scale |  |  |  |
| Model 1 | -0.08 (-0.24, 0.08) | -0.06 (-0.16, 0.05) | -0.01 (-0.07, 0.06) |
| Model 2 | -0.07 (-0.24, 0.10) | -0.04 (-0.14, 0.07) | 0.02 (-0.06, 0.09) |
| Model 3 | -0.06 (-0.23, 0.11) | -0.06 (-0.16, 0.05) | 0.01 (-0.07, 0.09) |
| Adjusted for near-road | -0.05 (-0.23, 0.12) | -0.06 (-0.17, 0.05) | 0.02 (-0.06, 0.10) |
| Adjusted for physical activity | -0.06 (-0.23, 0.11) | -0.06 (-0.16, 0.05) | 0.01 (-0.07, 0.09) |
| Adjusted for screen time | -0.05 (-0.22, 0.11) | -0.06 (-0.17, 0.05) | 0.01 (-0.07, 0.09) |

^a^ Model 1 includes adjustment for child sex, child age at outcome assessment. Model 2 includes adjustment for the variables in model 1 as well as adjustment for maternal education, household income adjusted for household size, maternal race, socioeconomic COI scale and education COI opportunity, urbanicity, and number of moves. Model 3 includes the variables in model 1 and 2 as well as maternal IQ, maternal depression, PRQ attachment score, maternal smoking during pregnancy, preterm birth, and is the primary model shown in Figure 1. Extended models with additional adjustments listed in the table also include covariates from model 3. Differences in CBCL scores (95% confidence intervals) are shown for a 0.1 unit higher NDVI, 10% higher tree cover, and living 500m closer to a park.

**Supplemental Table 2.** Associations with NDVI in multiple buffer sizes.

| **NDVI buffer size** ^a^ | **β (95% CI)** ^b^ |
| --- | --- |
| Internalizing score |  |
| 100m | -0.57 (-1.06, -0.09) |
| 300m | -0.66 (-1.26, -0.07) |
| 1000m | -0.63 (-1.27, 0.01) |
| Externalizing score |  |
| 100m | -0.08 (-0.60, 0.43) |
| 300m | -0.36 (-1.00, 0.27) |
| 1000m | -0.69 (-1.44, 0.06) |
| Attention problems |  |
| 100m | -0.07 (-0.21, 0.07) |
| 300m | -0.06 (-0.23, 0.10) |
| 1000m | -0.05 (-0.26, 0.15) |

^a^ NDVI was calculated as a weighted average across all residential locations in the year prior to the age 4-6 study visit.

^b^ Differences (95% confidence interval) in CBCL scores are shown per 0.1 unit higher NDVI. Linear regression models were adjusted for child sex, child age at outcome assessment, maternal education, household income adjusted for household size, maternal race, socioeconomic COI scale and education COI opportunity, urbanicity, residential stability, maternal IQ, maternal depression, PRQ attachment score, maternal smoking during pregnancy, and preterm birth.

**Supplemental** **Table 3.** Associations between distance to the nearest small, neighborhood, and community park and child behavior.

| **Park size ^a^** | **β (95% CI) ^b^** |
| --- | --- |
| Internalizing score |  |
| Small parks | 0.02 (-0.05, 0.08) |
| Neighborhood parks | 0.02 (-0.11, 0.15) |
| Community parks | 0.04 (-0.15, 0.23) |
| Externalizing score |  |
| Small parks | 0.04 (-0.04, 0.11) |
| Neighborhood parks | 0.03 (-0.14, 0.20) |
| Community parks | 0.12 (-0.09, 0.33) |
| Attention problems |  |
| Small parks | 0.02 (-0.01, 0.04) |
| Neighborhood parks | 0.01 (-0.04, 0.06) |
| Community parks | 0.05 (0, 0.11) |

^a^ Small parks were defined as those < 2 acres, neighborhood parks were defined as those between 2 and 20 acres, and community park were defined as parks >20 acres.

^b^ Models were adjusted for child sex, child age at outcome assessment, maternal education, household income adjusted for household size, maternal race, socioeconomic COI scale and education COI opportunity, urbanicity, residential stability, maternal IQ, maternal depression, PRQ attachment score, maternal smoking during pregnancy, and preterm birth. Regression coefficient is reported per 500m closer distance to the nearest park.

**Supplemental** **Table 4.** Association between green space and child CBCL scores among boys and girls.

|  | **NDVI**  β (95% CI) | **Tree cover**  β (95% CI) | **Park proximity**  β (95% CI) |
| --- | --- | --- | --- |
| Internalizing domain scores |  |  |  |
| Boys | -0.95 (-1.91, 0.01) | -0.30 (-0.77, 0.17) | -0.03 (-0.34, 0.27) |
| Girls | -0.40 (-1.07, 0.27) | -0.29 (-0.75, 0.18) | -0.12 (-0.40, 0.17) |
| Interaction p-value | 0.34 | 0.97 | 0.66 |
| Externalizing domain scores |  |  |  |
| Boys | -0.65 (-1.60, 0.30) | -0.43 (-0.95, 0.09) | -0.06 (-0.44, 0.33) |
| Girls | -0.09 (-0.89, 0.70) | -0.26 (-0.80, 0.28) | -0.04 (-0.35, 0.28) |
| Interaction p-value | 0.37 | 0.66 | 0.93 |
| Attention problems syndrome scale |  |  |  |
| Boys | -0.21 (-0.44, 0.02) | -0.09 (-0.23, 0.06) | -0.01 (-0.12, 0.10) |
| Girls | 0.07 (-0.16, 0.30) | -0.04 (-0.19, 0.11) | 0.04 (-0.06, 0.13) |
| Interaction p-value | 0.08 | 0.66 | 0.48 |

^b^ Models were adjusted for child sex, child age at outcome assessment, maternal education, household income adjusted for household size, maternal race, socioeconomic COI scale and education COI opportunity, urbanicity, residential stability, maternal IQ, maternal depression, PRQ attachment score, maternal smoking during pregnancy, and preterm birth. Differences in CBCL scores (95% confidence intervals) are shown for a 0.1 unit higher NDVI, 10% higher tree cover, and living 500m closer to a park.
